# Lifelong Caloric Restriction Increases Working Memory in Mice

**DOI:** 10.1371/journal.pone.0068778

**Published:** 2013-07-10

**Authors:** Angela Kuhla, Sophie Lange, Carsten Holzmann, Fabian Maass, Jana Petersen, Brigitte Vollmar, Andreas Wree

**Affiliations:** 1 Institute for Experimental Surgery, University of Rostock, Rostock, Germany; 2 Institute of Anatomy, University of Rostock, Rostock, Germany; 3 Institute of Medical Genetics, University of Rostock, Rostock, Germany; The Hebrew University Medical School, Israel

## Abstract

Caloric restriction (CR) is argued to positively affect general health, longevity and the normally occurring age-related reduction of cognition. This issue is well examined, but most studies investigated the effect of short-term periods of CR. Herein, 4 weeks old female mice were fed caloric restricted for 4, 20 and especially for 74 weeks. CR mice received 60% of food eaten by their ad libitum (AL) fed littermates, and all age-matched groups were behaviorally analyzed. The motor coordination, which was tested by rotarod/accelerod, decreased age-related, but was not influenced by the different periods of CR. In contrast, the age-related impairment of spontaneous locomotor activity and anxiety, both being evaluated by open field and by elevated plus maze test, was found aggravated by a lifelong CR. Measurement of cognitive performance with morris water maze showed that the working memory decreased age-related in AL mice, while a lifelong CR caused a better cognitive performance and resulted in a significantly better spatial memory upon 74 weeks CR feeding. However, a late-onset CR feeding in 66 weeks old mice did not ameliorate the working memory. Therefore, a lifelong CR seems to be necessary to improve working memory.

## Introduction

Aging affects all systems, but the brain seems to be particularly vulnerable to the action of negative, age-dependent factors. A gradual loss of memory functions and in this context of neuronal plasticity is one of the earliest and most widespread consequences of brain aging [1]. Thereby, the age-related loss of synaptic connection and attenuation of neuropil cause the subtle loss of cognitive skills leading to memory impairment [2], [3]. One possibility to reduce selective aspects of declined neuronal plasticity with aging is fitness training [4]. According to this, there is widely known that not only mental activity, but also aerobic exercise results in an enhancement of cognition [5]. Further, it is known that caloric restriction (CR) may also reduce neuronal damage and consequently offer protection against neurodegenerative diseases [6]. In addition Geng et al. [7], [8] describe in this context that CR in rats is beneficial for learning and memory as well as for social behavior and increased spontaneous locomotor activity. However, results on the protective effect of CR on aging-related cognitive decline in rodents are not consistent [9]. For example Bellush et al. [10] demonstrated that CR has no protective influence in age-dependent spatial learning, while Carter et al. [11] could show that CR resulted in an overall increase in physical activity. One reason for this discrepancy could be the age at which the CR is started. It has been shown that CR in rats results in significant life extension when started in young adulthood or earlier, but during middle age this response to CR is lost [9]. The findings on the effect of late-life CR in mice are also controversial. CR initiated in 12 or 19 months old mice decreased mortality and extended life [12], [13]. On the contrary Forster et al. [14] demonstrated that CR starting at age of 17 or 24 month increased the mortality of mice. Not only the studies, which examine the longevity in response to CR, are contradictory, but also the findings of behavior tests, which evaluated cognition and anxiety, are inconsistent [10], [15]. Furthermore, it is unclear how far the health-span effect of CR might extend into the life span. Thus, it is critical to determine whether longevity is associated with increase or limitation of cognitive capacity [11]. In addition, it is also conspicuously that not only the age but also the strain and the gender influence the effect of CR. Furthermore, the success of CR is dependent on the duration of CR [16], the intensity of food restriction [17], the behavioral testing and learning tasks as well as the age at which mice were tested [18]. All these parameters varied among the studies and make their results nearly incomparable [10]. With the present study, we focused on a lifelong CR and started with CR at weaning across middle age until very late age in order to maximize the reaction of the mice to CR.

## Materials and Methods

### Animals and Feeding

**Figure 1:**
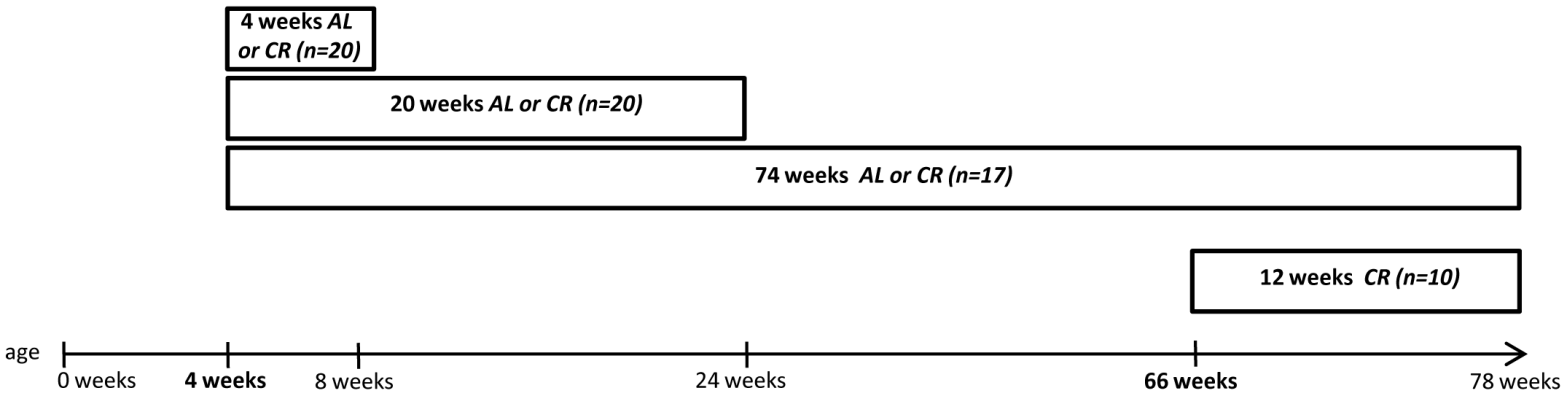
Experimental workflow. 4 weeks old AL mice were fed either ad libitum (AL) or caloric restricted (CR, 60% of ad libitum) for 4 weeks, 20 weeks or 74 weeks. An additional group of mice underwent a late-onset CR which started at the age of 66 weeks.

A total of 70 female mice (inbred strain C57BL/6, Charles River Wiga, Sulzfeld, Germany), 21 days old and weighing 14–16 g at the beginning of the experiments, were included in the present study. Being aware that animal behavior can considerably vary due to different environmental conditions, special attention was paid on standardized housing conditions: Up to the age of 28 days mice were housed in standard cages in groups of five animals in a temperature- and humidity-controlled room (21°C±3°C; 60%±6%) on a 12 h light/dark cycle (light on at 06.00) with free access to food and water under specific pathogen free (SPF) conditions. We used 4 weeks old C57BL/6 mice which were either ad libitum (AL) or caloric restricted (CR, 60% of ad libitum) fed for 4 weeks, 20 weeks or 74 weeks (Fig. 1) followed by behavioral tests. Thus, from day 29 onwards mice were divided into 6 groups: 4 weeks AL (n = 10) and CR (n = 10), 20 weeks AL (n = 10) and CR (n = 10), 74 weeks AL (n = 10) and CR (n = 10). The experimental protocol was approved by the local Animal Research Committee (Landesamt für Landwirtschaft, Lebensmittelsicherheit und Fischerei (LALLF) of the state Mecklenburg-Western Pomerania (LALLF M-V/TSD/7221.3–1.1-064/08) and all animals received humane care according to the German legislation on protection of animals and the Guide for the Care and Use of Laboratory Animals (NIH publication 86–23 revised 1985).

Mice were fed ad libitum (AL) with pelleted standard laboratory chow (Ssniff R/M-H 10 mm, Soest, Germany; cat. no. V154-000) and tap water and served as reference groups. Food intake of 5 AL fed mice housed per cage was measured once a week. From the daily eaten lab chow per AL fed mouse the CR fed mice received 60% once a day at 08∶00 [18]. AL feeding and caloric restriction lasted for 4, 20, and 74 weeks (Fig. 1). Induction of CR by feeding 40% fewer calories of that ad libitum-fed AL controls is a widely established model [17], [19].

In an additional experimental setup 66 weeks old mice (n = 10; LALLF M-V/TSD/7221.3-1.1-064/08) underwent a late-onset CR for a duration of 12 weeks (Fig. 1). Mice originated from our inbred C57BL/6 mouse colony (Charles River) and were kept under identical housing conditions as mentioned above.

### Behavioral Testing

**Figure 2:**
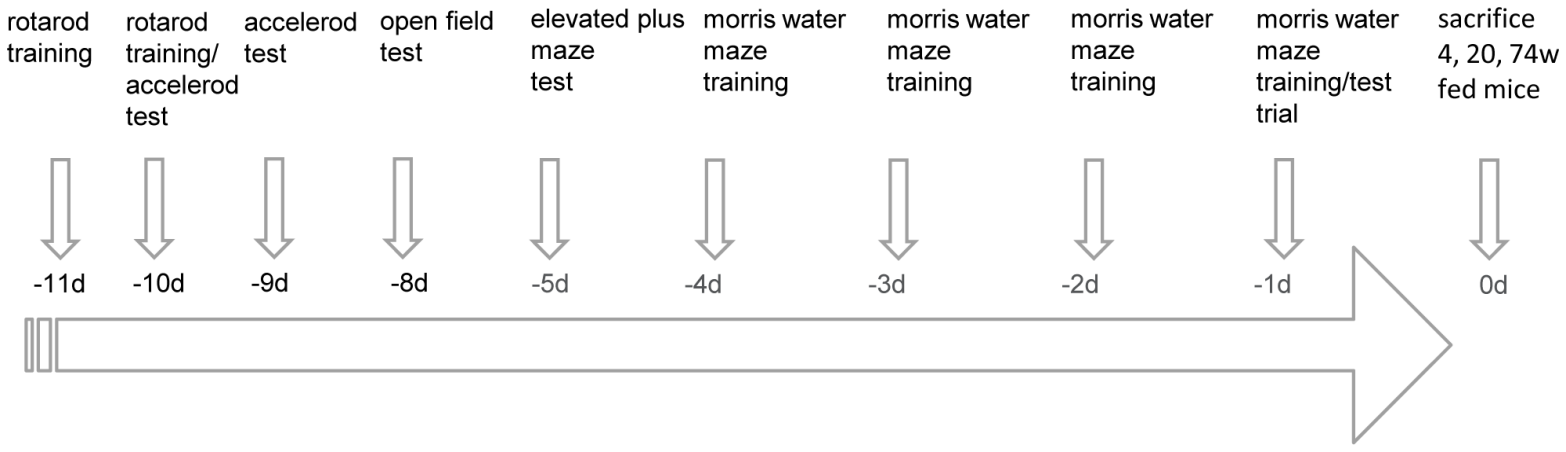
Timetable of the behavioral tests. The behavioral tests were started according to the given timetable (−11d) until sacrifice of animals (0d).

In each group, eleven days prior to sacrifice, testing of the AL and CR fed mice started according to the schedule shown in Fig. 2. Testing was performed during light phase (morris water maze) or during dark phase in the test room (rotarod and accelerod, open field, elevated plus maze).

### Rotarod/accelerod Test

The rotarod/accelerod test is widely used to evaluate motor skill learning, coordination, balance and ataxia [20], [21]. The accelerod test has been shown to be more sensitive than the rotarod test detecting motor function deficits and providing more consistent results [22]. Here, the accelerod system for mice (TSE-Systems, Bad Homburg, Germany) was used under standard room conditions. The rotating rod (30 mm diameter, 114 mm width) had a non-skid surface. Before accelorod testing, mice were trained 4 times a day on 2 consecutive days (−11d and −10d, Fig. 2) with a constant speed of 12 rpm for 120 s. The latency and frequency of slip offs from the rod within 120 s was recorded. During the 120 s training trials, mice were immediately replaced on the apparatus. Finally, a steady baseline level of performance was attained. When tested in the acceleration modus 4 times a day on 2 consecutive days (−10d and −9d, Fig. 2), the rotation speed continuously increased from 4 to 40 rpm within 5 min. Again, the time spent on the rod before falling off and the maximum speed level reached were recorded.

### Open Field Test

General locomotor activity and anxiety-like behavior of mice were evaluated in a square open field (OF) arena of 50×50 cm, placed inside an isolated box (TSE-Systems, Bad Homburg, Germany). Illumination of the open field was set to 300 Lux. Thirty min before the test started the animals were kept at dark-phase in the test room. Mice were tested once in the arena for 15 min (−8d, Fig. 2), while monitored online by a video camera placed inside the box and tracked using VideoMot2 Software (TSE Systems). The OF was divided in a peripheral and centre area by a grid in the tracking software: the arena consists of 16 quadrants, the 4 in the middle being defined as central. In between testing the individual mice the maze was carefully cleaned up with a wet towel. This test paradigm mimicked the natural conflict in mice between exploring a novel environment and avoiding an illuminated open area [21], [23]–[26]. The horizontal activity of the animals, their total migration distance, vertical activity and centre distance were recorded. Measures of anxiety-like behavior were the ratio of center distance to total distance and the defecation rate [25]. Mice were excluded from the OF test if they refused to move within 15 min.

### Elevated Plus Maze Test

The elevated plus maze (EPM) was used for exploring spontaneous and anxiety-like behavior [21], [27], [28]. The maze consisted of two open arms and two closed arms (arm length 300 mm, arm width 45 mm, wall height 150 mm, width of ledges 5 mm) arranged in a way that identical arms were placed opposite to each other. Arms emerged from a central platform (50×50 mm) of the entire apparatus raising 60 cm above the floor. Experiments were performed under dim light conditions (red photo bulb, 2 Lux) at −5d (Fig. 2). As for the OF test, 30 min before test start the animals were kept at dark-phase. For the single experiment mice were placed onto the central platform facing a closed arm and were monitored online by a video camera and tracked using VideoMot2 Software (TSE Systems) for 10 min. In between testing the individual mice the maze was carefully cleaned up with a wet towel. Anxiety-like behavior was evaluated through the parameter ratio of open arm entries to total arm entries. Since anxiolytic and anxiogenic effects can be confounded by changes in motor activity, locomotion was also evaluated as closed arm entries and total of arm entries. According to Dawson & Tricklebank [29] both parameters are considered to be the best indicators of locomotor activity of rodents placed on the elevated plus maze. Operational criterion for an arm entry was the presence of the whole body and four paws on the respective arm. In case of no or very little movement, animals were excluded.

### Morris Water Maze Test

The morris water maze (MWM) was performed as a task for measuring spatial reference memory. Mice under dim light conditions (indirect illumination, 3.0 Lux) at days −4d to −1 day were trained to locate a submerged hidden platform (11 cm diameter) in a fixed quadrant of a circular pool filled with opaque water (diameter of pool 107 cm, filled to a depth of 20 cm, water temperature 17°C, platform submerged 1 cm beneath the surface) [21]. For each trial (1×at −4d to familiarize with test apparatus, 8×at −3d, 8×at −2d, 4×−1d = 5 training blocks each consisting of 4 consecutive trials) mice are gently placed in the water, hind feet-first. On each trial, the starting position was randomized between four possible quadrant positions. The location of the platform remained constant throughout the whole training period. Each trial lasted until the animal located the platform; however, after maximal 60 s the mouse was guided to the platform. The latter was given a latency score of 60 s. Mice were allowed to rest 10 s on the escape platform, were then removed, dried with a towel and warmed under a heating lamp. After further 30 s the next trial started. The time to reach the platform (latency to escape) was recorded for each trial. On day −1, mice finally were trained four trials. All training trials were monitored. After training, the platform was removed from the pool and the mouse performed a 60 s probe trial. The start position of the probe trial was located opposite to the quadrant that had contained the platform. The swimming route and the number of times the mouse crossed each of the four possible platform positions (the previous platform position and 3 imaginary platform position in the other quadrants and platform crossings) was monitored online by a video camera and tracked using VideoMot2 Software (TSE Systems). Mice were excluded from the MWM if they did not find the platform at least once at the third day of training or if they refused to swim repeatedly [30].

### Statistical Analysis

Data were subjected to one- or two-way ANOVA with one between-subject factor (AL, CR) and with repeated measurements depending on the test used. The Holm-Sidak was used for post hoc comparisons. P<0.05 was considered significant for all tests throughout the study. In case of failed normality test, data were subjected to one- or two-way ANOVA on ranks. Dunn’s test or Tukey test was used for post hoc comparisons after ANOVA on ranks. Absolute values are given in means±SEM.

## Results

### Development of Body Weight is Slowed in Lifelong Caloric Restricted Fed Mice

**Figure 3:**
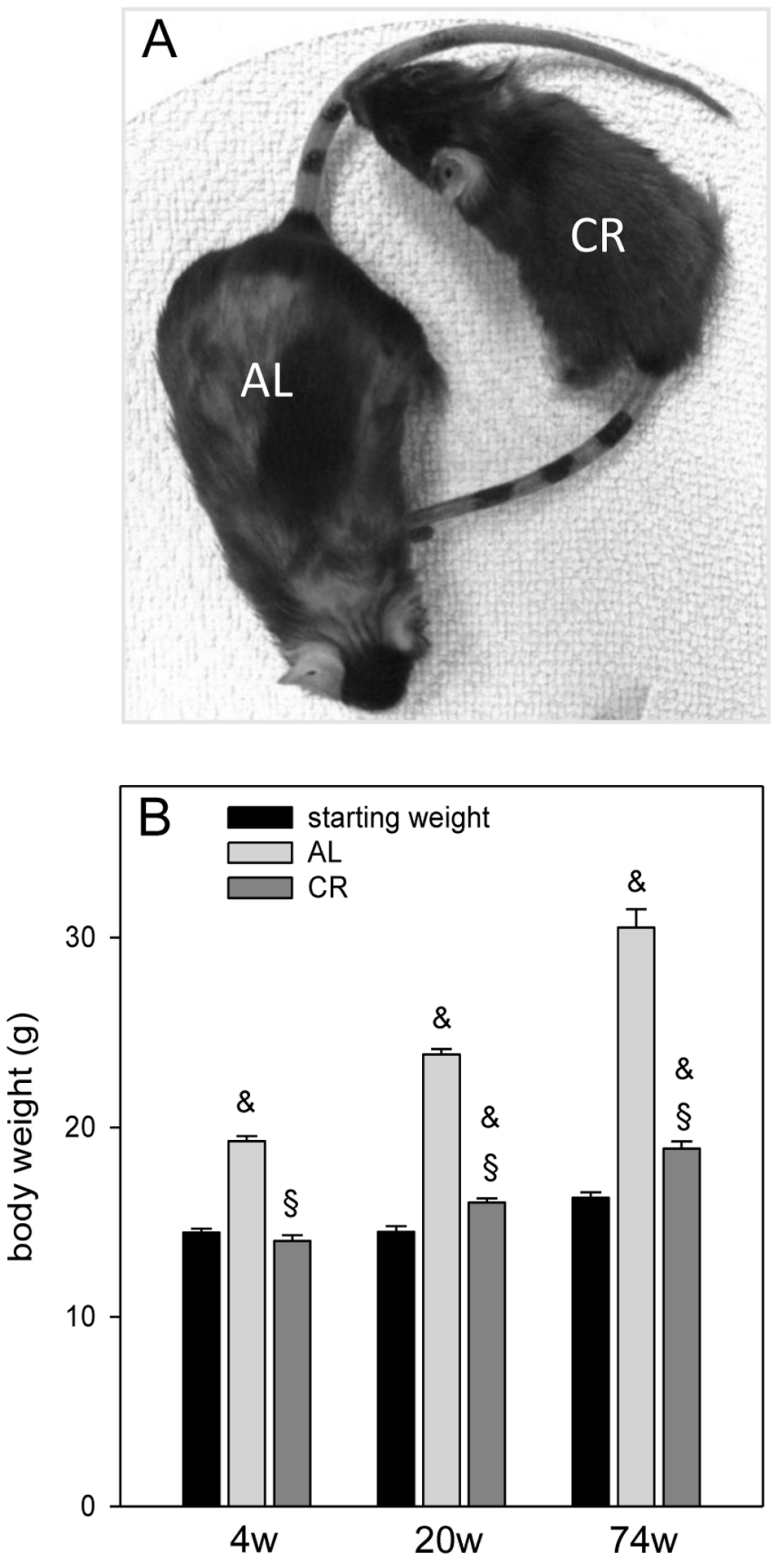
Appearance and body weight of mice. (A) Image of one 74 weeks ad libitum (AL) and one caloric-restricted (CR) fed C57BL/6J mouse. These mice exemplarily showed that in general CR mice were smaller in body size and had fewer external age-related signs as showing denser coat and barely greyed hair than the AL mice. (B) Starting weight (n_4w, 20w, 74w_ = 20, 20, 17) and body weight (g) of the 4 (4w), 20 (20w) and 74 (74w) weeks ad libitum (AL) and caloric-restricted fed mice were shown (AL: n_4w, 20w, 74w_ = 10, 10, 7; CR: n_4w, 20w, 74w_ = 10, 10, 10). In all AL fed mice, 20 and 74 weeks CR fed mice body weight was significantly increased when compared to their starting weight (4 weeks: F_1,28_ = 197.69, P<0.001; 20 weeks: F_1,28_ = 364.85, P<0.001; 74 weeks: F_1,22_ = 346.88, P<0.001). Compared with the age-matched AL fed mice the CR fed mice had significantly lower weights (4 weeks: F_1,18_ = 176.14, P<0.001; 20 weeks: F_1,18_ = 442.02, P<0.001; 74 weeks: F_1,15_ = 156.76, P<0.001). Values are given as mean±SEM; ANOVA, post-hoc pairwise comparison tests: ^&^P<0.05 vs. starting weight; ^§^P<0.001 vs. AL.

Generally, survival rate and health status were much better in the CR fed group. All CR fed mice reached the scheduled end of the experiments, whereas only 7 of 10 of AL fed mice lived for 78 weeks until sacrifice. Moreover, CR vs. AL fed mice were smaller in body size and had fewer external age-related signs as showing denser coat and barely greyed hair (Fig. 3A). The body weight of AL fed mice was significantly increased when compared to the starting weight (Fig. 3B) and continuously rose with aging as indicated by an increase up to 57% with duration of feeding. In contrast, lifelong CR feeding resulted in a slow but still detectable increase of body weight up to 34%. Compared with 4, 20 and 74 weeks AL fed mice, age-matched CR fed mice had significantly lower body weights (Fig. 3B).

### Accelerod Test - Motor Coordination Decreased Age-related and Basically was not Influenced by Lifelong Caloric Restriction

**Figure 4:**
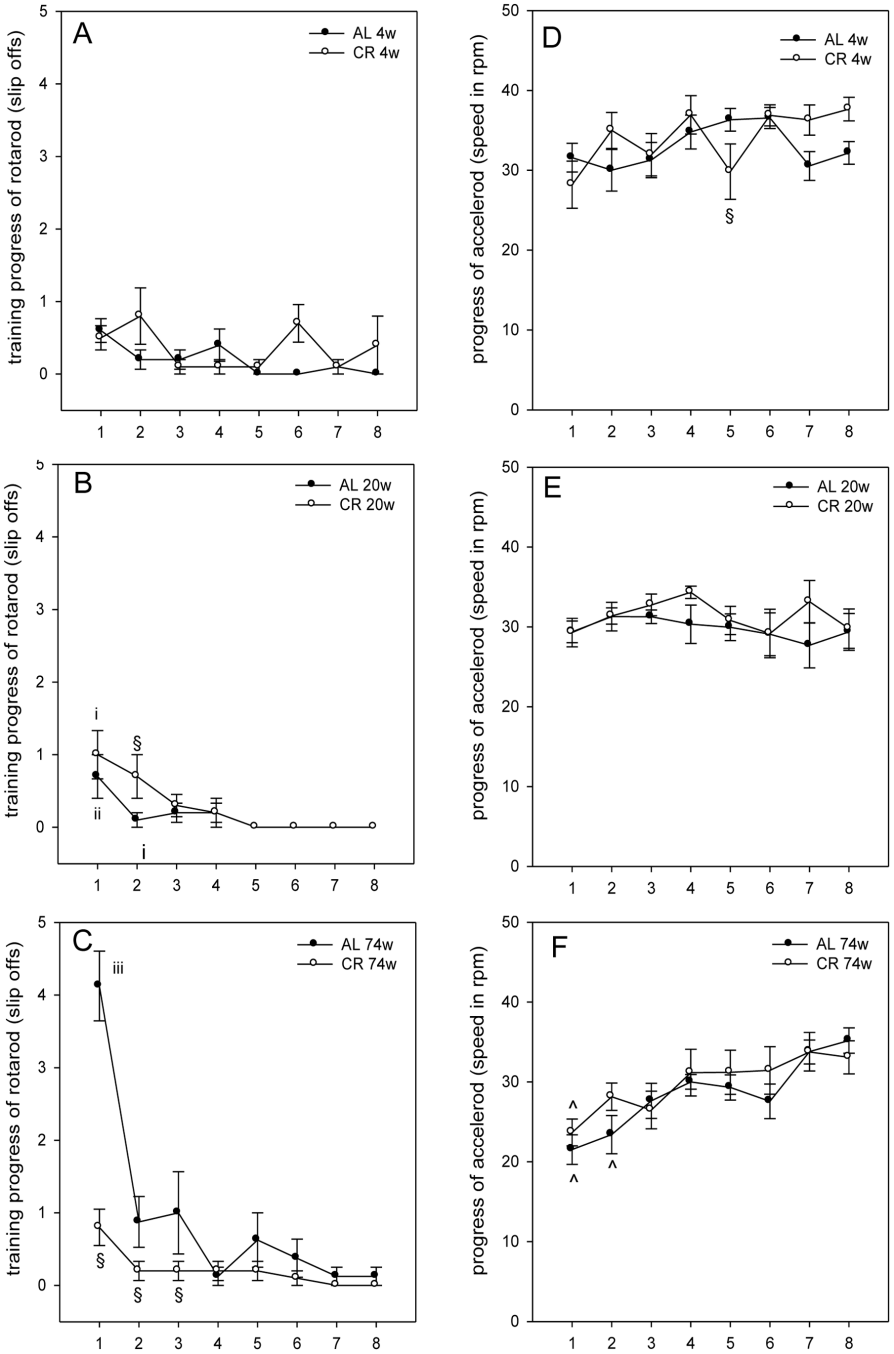
Motor coordination on the rotarod and accelerod test. (A–C) The slip offs of the mice (AL: n_4w, 20w, 74w_ = 10, 10, 8; CR: n_4w, 20w, 74w_ = 10, 10, 10) from the rotarod were measured on 8 training trials (x-axis 1–8). (A) There were no significant differences in falling downs from the rotarod detected between 4 weeks (4w) ad libitum (AL) and caloric-restricted (CR) fed mice (group: F_1,18_ = 2.373, P = 0.141; trial: F_1,7_ = 1.961, P = 0.066; interaction: F_1,7_ = 1.919, P = 0.072). (B) Two-way repeated measures ANOVA (group vs. trial) of 20 weeks groups slip offs showed only a statistical significance for the main factor trial, but not for main factor group and for interaction (group: F_1,18_ = 1.301, P = 0.269; trial: F_1,7_ = 8.210, P<0.001; interaction: F_1,7_ = 1.102, P = 0.366). (C) There are significant differences between 74 weeks AL and CR fed mice for training trial 1–3, but 74 weeks AL fed mice showed a fast improvement of their rotarod performance. 74 weeks CR fed mice exhibited no significant differences between the slip offs of trial 1–8. Two-way repeated measures ANOVA (group vs. trial) of 74 weeks groups slip offs revealed for the main variables group and trial as well as for interaction between these factors statistically significant differences (group: F_1,16_ = 20.453, P<0.001; trial: F_1,7_ = 24.527, P<0.001; interaction: F_1,7_ = 11.732, P<0.001; Fig. 4C). Values are given as mean±SEM; ANOVA, post-hoc pairwise comparison tests: ^§^P<0.05 vs. AL; ^i^P<0.05 vs. trial 5–8; ^ii^P<0.05 vs. trial 4–8; ^iii^P<0.05 vs. trial 2–8. (D–F) In the accelerod test the maximum speed (rpm) was measured in 8 test trials (x-axis 1–8) (AL: n_4w, 20w, 74w_ = 10, 10, 8; CR: n_4w, 20w, 74w_ = 10, 10, 10). Values are given as mean±SEM; ANOVA, post-hoc pairwise comparison tests: ^§^P<0.05 vs. AL; ^∧^P<0.05 vs. trial 7–8.

The balance and motor coordination of mice were measured with an accelerod system. All mice (4, 20, and 74 weeks AL and CR fed) habituated quickly to the rotating rod and attained an almost same steady baseline level in latency and frequency of slip offs during training (Fig. 4A–C). Noteworthy, 74 weeks AL vs. CR fed mice had significantly more slip offs at the beginning of the training, but showed a fast improvement of their rotarod performance (Fig. 4C).

Accelerod test demonstrated that 4 and 20 weeks AL and CR fed mice revealed no significant changes of maximum speed over the observation periods of 8 trials. Furthermore, no significant difference between 4 and 20 weeks AL vs. CR fed mice, except for test trial 5 in 4 weeks fed mice, was detected (Fig. 4D–E). Further, it could be demonstrated that with increasing speed both 74 weeks AL and CR fed mice exhibited a significantly better motor coordination over the test period without differing to each other (Fig. 4F).

### Open Field - Aging Decreased Spontaneous Locomotor Activity and Anxiety-like Behavior which is Aggravated with Lifelong Caloric Restriction

The OF test examined the locomotor activity and the anxiety-like behavior of mice. Total distance (cm during 15 min), a parameter of horizontal activity, was 8179±381, 8525±434, and 4765±419 for the 4, 20 and 74 weeks AL fed mice and 6879±416, 7194±483, and 4426±406 for the age-matched CR fed mice. These data revealed that the 4 weeks CR fed mice had a significantly lower horizontal activity when compared to age-matched AL fed mice (F_1,17_ = 5.321, P = 0.034; data not shown). All other AL vs. CR fed animals (20 and 74 weeks) revealed no significant differences of locomotor activity (20 weeks: F_1,18_ = 4.196, P = 0.055; 74 weeks: F_1,16_ = 0.329, P = 0.574). 74 weeks AL fed mice as well as age-matched CR fed mice moved significantly less in the OF when compared to 4 weeks fed animals reflecting an age-related reduction of exploratory and motor activity (AL: F_2,25_ = 23.190, P<0.001, CR: F_2,26_ = 12.186, P<0.001).

In the OF also rearing frequency during 15 min was detected. 4, 20 and 74 weeks AL fed mice reared 100.0±4.6, 53.5±3.9, and 32.2±6.1 and age-matched CR fed mice reared 73.7±7.1, 48.7±3.2, and 37.1±4.3. The rearing rate, a parameter of vertical activity, was significantly decreased in 4 weeks CR fed mice when compared to 4 weeks AL fed mice (F_1,18_ = 9.548, P = 0.006). The rearing events significantly decreased with duration of AL and CR feeding (AL: F_2,25_ = 50,787, P<0.001, CR: F_2,27_ = 13.110, P<0.001).

**Figure 5:**
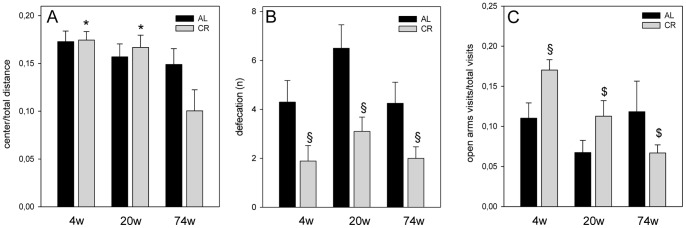
Locomotor activity and anxiety-like behavior in the open field (OF) and elevated plus maze (EPM). (A) Anxiety-like behavior was measured by the ratio of center distance to total distance in the OF. The ratio did not detect significant differences between ad libitum (AL) and caloric-restricted (CR) fed mice (AL: n_4w, 20w, 74w_ = 10, 10, 8; CR: n_4w, 20w, 74w_ = 9, 10, 10). However, 74 weeks (74w) vs. 4 weeks (4w) CR fed mice revealed a decreased ratio indicating a raised level of anxiety-like behaviour (F_2,26_ = 6.551, P = 0.005). Values are given as mean±SEM; ANOVA, post-hoc pairwise comparison tests: ^*^P<0.05 vs. 74w. (B) The defecation rate in OF, a parameter of anxiety-like behavior, was significantly higher in all ad libitum (AL) fed mice when compared to age-related caloric-restricted (CR) fed mice (4 weeks: F_1,17_ = 4.729, P = 0.044; 20 weeks: F_1,18_ = 9.175, P = 0.007; 74 weeks: F_1,16_ = 5.854, P = 0.028. Values are given as mean ± SEM; ANOVA, post-hoc pairwise comparison tests: ^§^P<0.05 vs. AL. (C) The ratio of open arms visits to total visits as a parameter of anxiety-like behavior was significantly increased only in 4 weeks CR fed mice when compared to age-matched AL fed mice (F_1,16_ = 6.115, P = 0.025) and significantly decreased upon lifelong CR (F_2,24_ = 10,713, P<0.001). Values are given as mean±SEM; ANOVA, post-hoc pairwise comparison tests: ^§^P<0.05 vs. AL, ^$^P<0.05 vs. 4w.

Anxiety-like behavior was measured by the ratio of center distance to total distance (Fig. 5A). The ratio did not detect any significant differences between AL and CR fed mice. Only duration of CR for 74 weeks caused a significant decrease of the ratio when compared to 4 weeks CR feeding indicating raised level of anxiety-like behavior (Fig. 5A).

The defecation rate, serving also as a parameter of anxiety-like behavior, was significantly higher in all AL fed mice when compared to age-related CR fed mice (Fig. 5B).

### Elevated Plus Maze – Lifelong Caloric Restriction Slightly Influenced Locomotor Activity and Emotionality

We performed the EPM test to confirm the differences in anxiety-like behavior as well as motor and exploratory activity which was displayed in the OF test. Activity was measured by determining the number of total visits of the 4, 20 and 74 weeks AL fed mice, which were 81.80±3.40, 70.50±4.28, and 66.75±6.31. Age-matched CR fed mice revealed number of total visits of 77.63±5.22, 50.50±4.33, and 50.33±2.76. 20 weeks and 74 weeks AL fed mice moved significantly more than age-related CR fed mice (20 weeks: F_1,18_ = 10.782, P = 0.004; 74 weeks: F_1,15_ = 6.160, P = 0.025). While AL fed mice did not reveal an age-related decline of motor activity, CR fed mice showed a significant decrease of motor activity (AL: F_2,25_ = 2.904, P = 0.073, CR: F_2,24_ = 13.281, P<0.001).

The ratio of open arms visits to total visits as a parameter of anxiety-like behavior was found significantly increased only in 4 weeks CR fed mice when compared to age-related AL fed mice (Fig. 5C), but significantly decreased over duration of CR (Fig. 5C). These results showed that 4 weeks CR fed mice, who spent more time in open arms, exhibited a lower anxiety-like behavior level as age-related AL fed mice. However, there was also a rise of anxiety-like behavior by the extension of duration of CR (20 and 74 weeks), which we did not observe in the AL fed mice. This observation confirms the results of the OF test.

### Morris Water Maze – Lifelong Caloric Restriction Increased Working Memory

**Figure 6:**
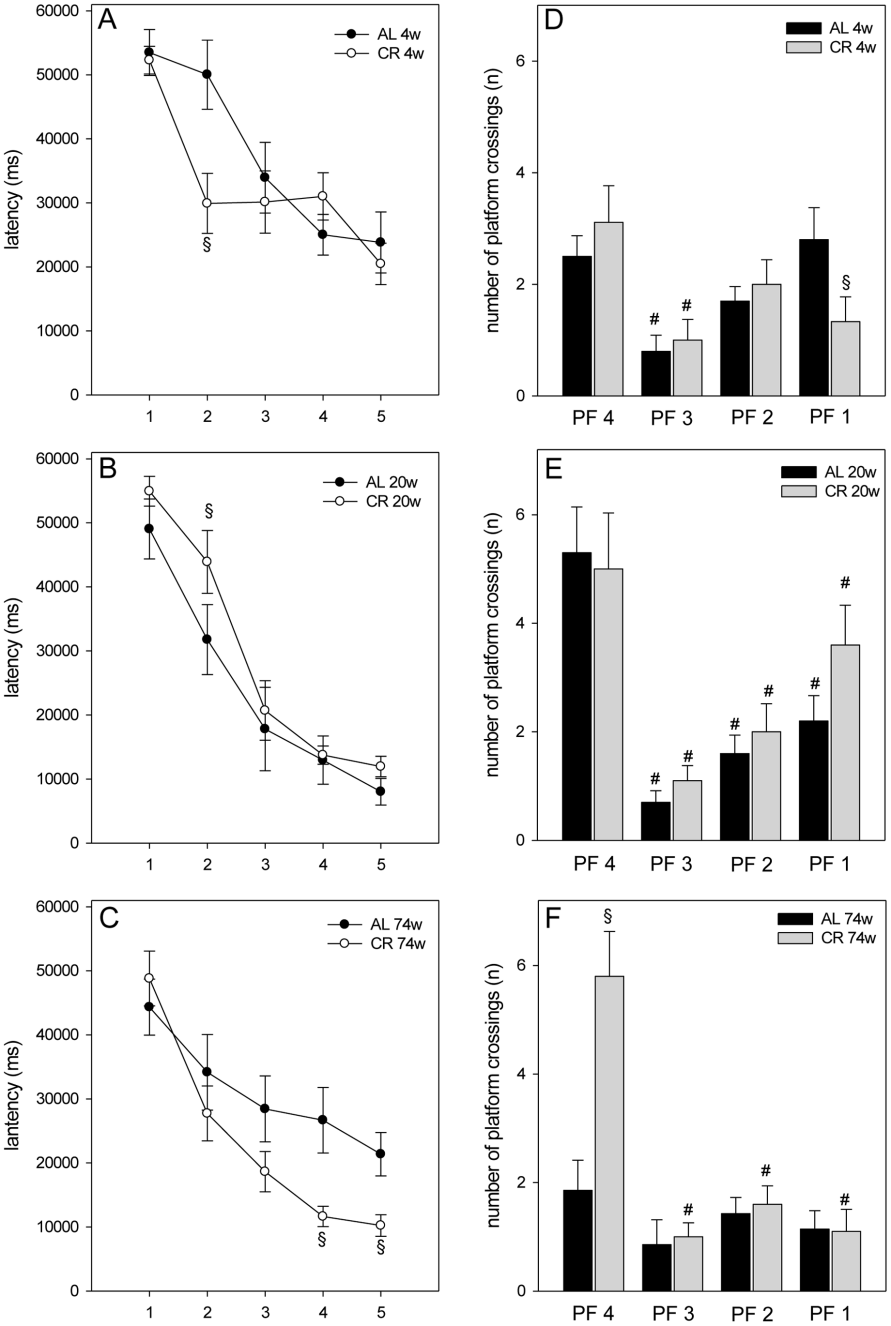
Spatial learning in morris water maze. (A–C) The escape latencies (in ms) of the training were displayed in five blocks (x-axis 1–5). One block consisted of four consecutive trials. The escape latencies decreased throughout the training days. (A–B) For the 4 (4w) and 20 weeks (20w) AL and CR fed groups two-way ANOVA for repeated measures (group vs. block) revealed no significance for the main factor group (4 weeks: F_1,17_ = 0.934, P = 0.347; 20 weeks: F_1,18_ = 1.523, P = 0.233), but a significant effect for the main factor block (F_1,4_ = 29.753, P<0.001; F_1,4_ = 62.468, P<0.001). The interaction between these variables is significant between 4 weeks AL and CR fed mice (F_1,4_ = 4.807, P = 0.002), but no significance could be shown for interaction of 20 weeks fed groups (F_1,4_ = 0.918, P = 0.458). Two-way ANOVA for repeated measures (group vs. block) of the 74 weeks (74w) AL and CR fed mice indicated a significant effect for the main factors (group: F_1,15_ = 4.816, P = 0.044; block: F_1,4_ = 25.078, P<0.001), but there was no statistically significant interaction between group and block (F_1,4_ = 2.312, P = 0.068). Subsequent Holm-Sidak tests with an overall significance level of P = 0.05 showed only significant differences of escape latency in block 2 respectively between 4 and 20 weeks AL and CR fed mice (Fig. 6A–B). (C) Between 74 weeks AL and CR fed groups there were significant differences of escape latency in blocks 4 and 5. Values are given as mean±SEM; ANOVA, post-hoc pairwise comparison tests: ^§^P<0.05 vs. AL. (D–F In this test trial, the number of platform crossings during 60 s was measured. (D and E) Two-way ANOVA of the 4 and 20 weeks fed groups (group vs. platform) showed no significances for the main factor group and for interaction between group and platform (4 weeks: group: F_1,72_ = 0.536, P = 0.466; interaction: F_1,3_ = 2.249, P = 0.090; 20 weeks: group: F_1,72_ = 1.188, P = 0.279; interaction: F_1,3_ = 0.644, P = 0.589). Values are given as mean±SEM; ANOVA, post-hoc pairwise comparison tests: ^#^P<0.05 vs. PF4; ^§^P<0.05 vs. AL. (F) The 74 weeks AL fed mice (n = 7) did not show significantly more crossings of PF4. Values are given as mean±SEM; ANOVA, post-hoc pairwise comparison tests: ^#^P<0.05 vs. PF4; ^§^P<0.05 vs. AL.

Mice were tested in the spatial reference memory version of the morris water maze. AL and CR fed mice did not show significant differences in their swimming performances which were determined by measurement of the swimming speed during the test trial. The escape latencies were displayed in five blocks. One block consisted of four consecutive trials. The escape latencies decreased throughout the training days. Thus, all mice were able to learn the task (Fig. 6A–C). While 4 and 20 weeks fed mice of both feeding groups revealed almost comparable escape latency, CR feeding for 74 weeks resulted in a significantly lower escape latency which was found for block 4 and 5 (Fig. 6C). Thus, a lifelong CR caused a better training performance.

All mice were exercised to the platform 4 (PF4) during training. Finally, the platform was removed and the number of platform crossings during 60 s was measured. 4 weeks fed mice of both feeding groups did not remember the PF4 position (Fig. 6D). In contrast, duration of both AL and CR for 20 weeks resulted in a better memory as indicated by the higher number of PF4 crossings (Fig. 6E). Further, 20 weeks AL and CR fed mice crossed PF4 significantly more often than PF1-3, showing no intergroup differences (Fig. 6E). The most striking differences between the AL and CR fed mice were found in the number of platform crossings of 74 weeks fed mice. While 74 weeks AL fed mice did not show significant differences in crossings of PF4 when compared to PF 1-3, the age-matched CR fed mice significantly more often crossed PF4 than PF1-3 with 3-fold higher values when compared to AL fed mice (Fig. 6F).

### Body Weight and Behavioral Tests – Influence of Late-onset CR in 66 Weeks Old Mice

These mice revealed a body weight of up to 30.3±0.94 g when CR feeding started, and a body weight of 19.8±0.7 g upon 12 weeks CR feeding (i.e. reduction of about 35%; F_1, 14_ = 81,408; P<0.001). Late-onset CR did not influence -similarly to lifelong CR- the motor coordination (tested by accelerod; data not shown). In addition, late-onset CR in advanced age had no effect on locomotor activity and anxiety-like behavior (data not shown). While a lifelong CR caused an increased working memory (tested by morris water maze), late-onset CR feeding in 66 weeks old mice resulted in a similar working memory as observed in 74 weeks AL fed mice (data not shown). A lifelong CR seems to be necessary to improve working memory, because mice with late-onset CR did not show improved spatial learning as lifelong CR mice did.

## Discussion

The major finding of the present study is that a lifelong CR aggravated the age-related loss of spontaneous motor activity and the increase of anxiety-like behavior. The motor coordination is also affected with aging but not further influenced by CR. Of utmost interest is the novel finding that lifelong CR however increases spatial learning and working memory.

Age-related impairments of cognitive and motor capacity have been linked to a number of deleterious morphologic and functional changes involving different parts of the brain that are normally associated with such functions [31]–[33]. In this context the majority of studies focused on the cognitive decline accompanied with aging [10], [31], [34]. These cognitive deficits have been shown to be partly reversed by CR [35]. However, there is controversy regarding the effects of dietary regimen on behavior [36], [37]. Levay et al. [37] reported that in rats the behavioral consequence of CR is a decrease of anxiety-like behavior whereas Jahng et al. [36] showed that chronic CR in young rats may lead to depressive and/or anxiety-like behavior disorders, most probably via dysregulation of the cerebral serotonin neurotransmitter system. Further studies also demonstrated that dieting, i.e. intentional caloric restriction to reduce body weight, is associated with the development of eating disorders and depression [38]. Due to the fact that depression is usually linked to anxiety, the present study shows that lifelong CR gradually increased anxiety-like behavior, as reflected by the declining number of open arms visits in the EPM test. This finding is in line with the report of Yamamoto et al. [15] demonstrating that CR affects behavior by an increase of dopamine- and cAMP-regulated phosphoprotein (Darpp-32) in the amygdala [15]. Further on, Tomiyama et al. showed that low calorie dieting increases cortisol, a psychological stress indicator [39]. The present study confirms the chronic CR-induced aggravation of anxiety-like behavior and extends the current knowledge [10], [15] in that CR reduces spontaneous motor activity which can be interpreted also as increased anxiety-like behavior. However, it should be kept in mind that CR fed mice may have limited energy stores which could lead to reduced motor activity. Further evidence for decreased CR-related energy availability and thus less motor activity is given by a low defecation rate. On the contrary, other studies [11], [40] described that CR results in an increase of physical activity which explains with hyperactivity in response to CR-associated stress [40].

Aging is characterized by oxidative stress damage within the cerebellum and the cerebral cortex [41] that consequently leads not only to less motor activity but also to a decline of motor coordination [42]. This finding might explain why in the present study presenescent AL fed mice fall down 4-times more from the rod at the beginning of the training compared to younger AL fed animals. Though a lifelong CR vs. AL feeding caused initially a significantly better motor coordination (Fig. 4C), this advance is lost with training because all mice regardless of age or nutrition behaved comparably well on the accelerod (Fig. 4D–F). Thus, it can be concluded that lifelong CR as performed in the present study, is not beneficial for motor coordination. One explanation is probably that CR has not the ability to delay the age-related shrinkage of striatal volume, and thus does not prevent the alteration in the integrity of dopaminergic projection to the striatum [43].

Spatial learning ability is impaired with age and associated with compromised hippocampal function [44]. While Bellush and colleagues [10] showed that a lifelong CR does not prevent age-related impairments in spatial learning using a morris water maze task, the present study could demonstrate that a lifelong CR increased spatial learning ability, similarly as found by other groups [45], [46]. In contrast, a late-onset and short-term CR did not improve spatial learning. These results are supported by the report of Minor and colleagues [47], which could demonstrate that a short-term CR of 8 weeks has no effect on memory enhancement. However, it has to be stated that the current data of the late-onset CR mice rather serve as pilot data and were not paralleled by an additional control group of 10 animals in a randomized fashion. Based on this, the findings did not undergo statistical analysis and have therefore to be interpreted with caution. Nevertheless, the failure of late-onset CR to improve spatial learning is sustained by others showing that acute dietary restriction did not exhibit a significantly better behavioural tasks testing memory [46].

Due to the fact that CR can stimulate the production of new neurons from stem cells (neurogenesis) and enhance synaptic plasticity due to an amelioration of the age-related decline in synaptophysin levels [44] we assume that the better spatial memory might be the consequence of CR-induced regenerative processes in the hippocampus. Moreover, it is known that ketone bodies are neuroprotective [48]–[50] and enhance memory [51]. In line with this, we could observe a significant increase of ketogenesis in lifelong CR fed mice (unpublished own data), and therefore assume, that this metabolic adaptation might have contributed to the improved working memory. In summary, a lifelong CR, i.e. starting at the age of weaning until presenescence, reduces the normal age-related decline in cognitive performance. However, it should be considered that CR indeed is beneficial for learning but also increases the risk of anxiety-like behavior.

## References

[1] Bergado JA, Almaguer W (2002) Aging and synaptic plasticity: a review. Neural Plast 9: 217–232. 12959152 10.1155/NP.2002.217 PMC2565407

[2] Terry RD, Katzman R (2001) Life span and synapses: will there be a primary senile dementia? Neurobiol Aging 22: 347–348. 11378236 10.1016/s0197-4580(00)00250-5

[3] Hof PR, Morrison JH (2004) The aging brain: morphomolecular senescence of cortical circuits. Trends Neurosci 27: 607–613. 15374672 10.1016/j.tins.2004.07.013

[4] Churchill JD, Galvez R, Colcombe S, Swain RA, Kramer AF, et al (2002) Exercise, experience and the aging brain. Neurobiol Aging 23: 941–955. 12392797 10.1016/s0197-4580(02)00028-3

[5] Erickson KI, Voss MW, Prakash RS, Basak C, Szabo A, et al (2011) Exercise training increases size of hippocampus and improves memory. Proc Natl Acad Sci U S A 108: 3017–3022. 21282661 10.1073/pnas.1015950108 PMC3041121

[6] Gillette-Guyonnet S, Vellas B (2008) Caloric restriction and brain function. Curr Opin Clin Nutr Metab Care 11: 686–692. 18827571 10.1097/MCO.0b013e328313968f

[7] Geng YQ, Guan JT, Xu MY, Xu XH, Fu YC (2007) Behavioral study of calorie-restricted rats from early old age. Conf Proc IEEE Eng Med Biol Soc 2007: 2393–2395. 18002475 10.1109/IEMBS.2007.4352809

[8] Geng YQ, Li TT, Liu XY, Li ZH, Fu YC (2011) SIRT1 and SIRT5 activity expression and behavioral responses to calorie restriction. J Cell Biochem 112: 3755–3761. 21826711 10.1002/jcb.23315

[9] Masoro EJ (2006) Dietary restriction-induced life extension: a broadly based biological phenomenon. Biogerontology 7: 153–155. 16732403 10.1007/s10522-006-9015-0

[10] Bellush LL, Wright AM, Walker JP, Kopchick J, Colvin RA (1996) Caloric restriction and spatial learning in old mice. Physiol Behav 60: 541–547. 8840916 10.1016/s0031-9384(96)80029-1

[11] Carter CS, Leeuwenburgh C, Daniels M, Foster TC (2009) Influence of calorie restriction on measures of age-related cognitive decline: role of increased physical activity. J Gerontol A Biol Sci Med Sci 64: 850–859. 19420296 10.1093/gerona/glp060 PMC2709546

[12] Pugh TD, Oberley TD, Weindruch R (1999) Dietary intervention at middle age: caloric restriction but not dehydroepiandrosterone sulfate increases lifespan and lifetime cancer incidence in mice. Cancer Res 59: 1642–1648. 10197641

[13] Dhahbi JM, Kim HJ, Mote PL, Beaver RJ, Spindler SR (2004) Temporal linkage between the phenotypic and genomic responses to caloric restriction. Proc Natl Acad Sci U S A 101: 5524–5529. 15044709 10.1073/pnas.0305300101 PMC397416

[14] Forster MJ, Morris P, Sohal RS (2003) Genotype and age influence the effect of caloric intake on mortality in mice. FASEB J 17: 690–692. 12586746 10.1096/fj.02-0533fje PMC2839882

[15] Yamamoto Y, Tanahashi T, Kawai T, Chikahisa S, Katsuura S, et al (2009) Changes in behavior and gene expression induced by caloric restriction in C57BL/6 mice. Physiol Genomics 39: 227–235. 19737990 10.1152/physiolgenomics.00082.2009

[16] Hashimoto T, Watanabe S (2005) Chronic food restriction enhances memory in mice–analysis with matched drive levels. Neuroreport 16: 1129–1133. 15973161 10.1097/00001756-200507130-00019

[17] Deng L, Wu ZN, Han PZ (2009) Effects of different levels of food restriction on passive-avoidance memory and the expression of synapsin I in young mice. Int J Neurosci 119: 291–304. 19125380 10.1080/00207450802328250

[18] Means LW, Higgins JL, Fernandez TJ (1993) Mid-life onset of dietary restriction extends life and prolongs cognitive functioning. Physiol Behav 54: 503–508. 8415944 10.1016/0031-9384(93)90243-9

[19] Mouton PR, Chachich ME, Quigley C, Spangler E, Ingram DK (2009) Caloric restriction attenuates amyloid deposition in middle-aged dtg APP/PS1 mice. Neurosci Lett 464: 184–187. 19699265 10.1016/j.neulet.2009.08.038 PMC2748166

[20] Jones BJ, Roberts DJ (1968) The quantiative measurement of motor inco-ordination in naive mice using an acelerating rotarod. J Pharm Pharmacol 20: 302–304. 4384609 10.1111/j.2042-7158.1968.tb09743.x

[21] Karl T, Pabst R, von Hörsten S (2003) Behavioral phenotyping of mice in pharmacological and toxicological research. Exp Toxicol Pathol 55: 69–83. 12940631 10.1078/0940-2993-00301

[22] Bogo V, Hill TA, Young RW (1981) Comparison of accelerod and rotarod sensitivity in detecting ethanol- and acrylamide-induced performance decrement in rats: review of experimental considerations of rotating rod systems. Neurotoxicology 2: 765–787. 7200586

[23] DeFries JC, Hegmann JP, Weir MW (1966) Open-field behavior in mice: evidence for a major gene effect mediated by the visual system. Science 154: 1577–1579. 5924928 10.1126/science.154.3756.1577

[24] Denenberg VH (1969) Open-field behavior in the rat: what does it mean? Ann N Y Acad Sci 159: 852–859. 5260302 10.1111/j.1749-6632.1969.tb12983.x

[25] Eikelis N, Van Den Buuse M (2000) Cardiovascular responses to open-field stress in rats: sex differences and effects of gonadal hormones. Stress 3: 319–334. 11342397 10.3109/10253890009001137

[26] Prut L, Belzung C (2003) The open field as a paradigm to measure the effects of drugs on anxiety-like behaviors: a review. Eur J Pharmacol 463: 3–33. 12600700 10.1016/s0014-2999(03)01272-x

[27] Lister RG (1987) The use of a plus-maze to measure anxiety in the mouse. Psychopharmacology (Berl) 92: 180–185. 3110839 10.1007/BF00177912

[28] Rodgers RJ, Dalvi A (1997) Anxiety, defence and the elevated plus-maze. Neurosci Biobehav Rev 21: 801–810. 9415905 10.1016/s0149-7634(96)00058-9

[29] Dawson GR, Tricklebank MD (1995) Use of the elevated plus maze in the search for novel anxiolytic agents. Trends Pharmacol Sci 16: 33–36. 7762079 10.1016/s0165-6147(00)88973-7

[30] Holzmann C, Dräger D, Mix E, Hawlitschka A, Antipova V, et al (2012) Effects of intrastriatal botulinum neurotoxin A on the behaviour of Wistar rats. Behav Brain Res 234: 107–116. 22728288 10.1016/j.bbr.2012.06.008

[31] Gage FH, Kelly PA, Björklund A (1984) Regional changes in brain glucose metabolism reflect cognitive impairments in aged rats. J Neurosci 4: 2856–2865. 6502208 10.1523/JNEUROSCI.04-11-02856.1984 PMC6564735

[32] Gallagher M, Burwell RD (1989) Relationship of age-related decline across several behavioral domains. Neurobiol Aging 10: 691–708. 2628781 10.1016/0197-4580(89)90006-7

[33] Ingram DK (1988) Complex maze learning in rodents as a model of age-related memory impairment. Neurobiol Aging 9: 475–485. 3062459 10.1016/s0197-4580(88)80101-5

[34] Barnes CA (1979) Memory deficits associated with senescence: a neurophysiological and behavioral study in the rat. J Comp Physiol Psychol 93: 74–104. 221551 10.1037/h0077579

[35] Wadden TA, Stunkard AJ (1986) Controlled trial of very low calorie diet, behavior therapy, and their combination in the treatment of obesity. J Consult Clin Psychol 54: 482–488. 3528252 10.1037//0022-006x.54.4.482

[36] Jahng JW, Kim JG, Kim HJ, Kim BT, Kang DW, et al (2007) Chronic food restriction in young rats results in depression- and anxiety-like behaviors with decreased expression of serotonin reuptake transporter. Brain Res 1150: 100–107. 17383614 10.1016/j.brainres.2007.02.080

[37] Levay EA, Govic A, Penman J, Paolini AG, Kent S (2007) Effects of adult-onset calorie restriction on anxiety-like behavior in rats. Physiol Behav 92: 889–896. 17673267 10.1016/j.physbeh.2007.06.018

[38] Wooley SC, Garner DM (1991) Obesity treatment: the high cost of false hope. J Am Diet Assoc 91: 1248–1251. 1918744

[39] Tomiyama AJ, Mann T, Vinas D, Hunger JM, Dejager J, et al (2010) Low calorie dieting increases cortisol. Psychosom Med 72: 357–364. 20368473 10.1097/PSY.0b013e3181d9523c PMC2895000

[40] Smith LK, Metz GA (2005) Dietary restriction alters fine motor function in rats. Physiol Behav 85: 581–592. 16045945 10.1016/j.physbeh.2005.06.013

[41] Forster MJ, Dubey A, Dawson KM, Stutts WA, Lal H, et al (1996) Age-related losses of cognitive function and motor skills in mice are associated with oxidative protein damage in the brain. Proc Natl Acad Sci U S A 93: 4765–4769. 8643477 10.1073/pnas.93.10.4765 PMC39353

[42] Fahlström A, Yu Q, Ulfhake B (2011) Behavioral changes in aging female C57BL/6 mice. Neurobiol Aging 32: 1868–1880. 20005598 10.1016/j.neurobiolaging.2009.11.003

[43] Matochik JA, Chefer SI, Lane MA, Roth GS, Mattison JA, et al (2004) Age-related decline in striatal volume in rhesus monkeys: assessment of long-term calorie restriction. Neurobiol Aging 25: 193–200. 14749137 10.1016/s0197-4580(03)00092-7

[44] Adams MM, Shi L, Linville MC, Forbes ME, Long AB, et al (2008) Caloric restriction and age affect synaptic proteins in hippocampal CA3 and spatial learning ability. Exp Neurol 211: 141–149. 18342310 10.1016/j.expneurol.2008.01.016 PMC2805131

[45] Geng YQ, Guan JT, Xu MY, Xu XH, Fu YC (2007) Behavioral study of calorie-restricted rats from early old age. Conf Proc IEEE Eng Med Biol Soc. 2007: 2393–2395. 10.1109/IEMBS.2007.4352809 18002475

[46] Hashimoto T, Watanabe S (2005) Chronic food restriction enhances memory in mice–analysis with matched drive levels. Neuroreport. 16: 1129–1133. 10.1097/00001756-200507130-00019 15973161

[47] Minor RK, Villarreal J, McGraw M, Percival SS, Ingram DK, et al (2008) Calorie restriction alters physical performance but not cognition in two models of altered neuroendocrine signaling. Behav Brain Res. 189: 202–211. 10.1016/j.bbr.2007.12.030 18291538

[48] Noh HS, Kim YS, Choi WS (2008) Neuroprotective effects of the ketogenic diet. Epilepsia 49: 120–123. 19049608 10.1111/j.1528-1167.2008.01855.x

[49] Kwon YS, Jeong SW, Kim DW, Choi ES, Son BK (2008) Effects of the ketogenic diet on neurogenesis after kainic acid-induced seizures in mice. Epilepsy Res 78: 186–194. 18201870 10.1016/j.eplepsyres.2007.11.010

[50] Maalouf M, Rho JM, Mattson MP (2009) The neuroprotective properties of calorie restriction, the ketogenic diet, and ketone bodies. Brain Res Rev 59: 293–315. 18845187 10.1016/j.brainresrev.2008.09.002 PMC2649682

[51] Krikorian R, Shidler MD, Dangelo K, Couch SC, Benoit SC, et al (2012) Dietary ketosis enhances memory in mild cognitive impairment. Neurobiol Aging 33: 19–27. 10.1016/j.neurobiolaging.2010.10.006 PMC3116949 21130529

